# Variability in Physiological Traits Reveals Boron Toxicity Tolerance in Aegilops Species

**DOI:** 10.3389/fpls.2021.736614

**Published:** 2021-10-29

**Authors:** Mohd. Kamran Khan, Anamika Pandey, Mehmet Hamurcu, Zuhal Zeynep Avsaroglu, Merve Ozbek, Ayse Humeyra Omay, Fevzi Elbasan, Makbule Rumeysa Omay, Fatma Gokmen, Ali Topal, Sait Gezgin

**Affiliations:** ^1^Department of Soil Science and Plant Nutrition, Faculty of Agriculture, Selcuk University, Konya, Turkey; ^2^Department of Field Crops, Faculty of Agriculture, Selcuk University, Konya, Turkey

**Keywords:** Aegilops, alien introgression, boron toxicity, genetic resources, genetic variation, stress tolerance, wheat, wild species

## Abstract

Boron (B) is an important micronutrient required for the normal growth and development of plants. However, its excess in the soil causes severe damage to plant tissues, which affects the final yield. Wheat, one of the main staple crops, has been reported to be largely affected by B toxicity stress in arid and semi-arid regions of the world. The prevalence of B toxicity stress can be addressed by utilizing wild wheat genotypes with a variant level of stress tolerance. Wild wheat relatives have been identified as a prominent source of several abiotic stress-tolerant genes. However, Aegilops species in the tertiary gene pool of wheat have not been well exploited as a source of B toxicity tolerance. This study explores the root and shoot growth, proline induction, and extent of lipid peroxidation in 19 Aegilops accessions comprising 6 different species and the B-tolerant check wheat cultivar Bolal 2973 grown under Control (3.1 μM B), toxic (1 mM B), and highly toxic (10 mM B) B stress treatment. B toxicity stress had a more decisive impact on growth parameters as compared to the malondialdehyde (MDA) and proline content. The obtained results suggested that even the genotypes with high shoot B (SB) accumulation can be tolerant to B toxicity stress, and the mechanism of B redistribution in leaves should be studied in detail. It has been proposed that the studied Aegilops accessions can be potentially used for genetically improving the B toxicity-tolerance trait due to a high level of variation in the response toward high B toxicity. Though a number of accessions showed suppression in the root and shoot growth, very few accessions with stress adaptive plasticity to B toxicity stress leading to an improvement of shoot growth parameters could be determined. The two accessions, *Aegilops biuncialis* accession TGB 026219 and *Aegilops columnaris* accession TGB 000107, were identified as the potential genotypes with B toxicity stress tolerance and can be utilized for developing a pre-breeding material in B tolerance-based breeding programs.

## Introduction

Boron (B) toxicity is a critical abiotic stress condition that has a detrimental effect on plant growth (Landi et al., 2019), thus limiting the agricultural yield in the different parts of the world, especially arid and semi-arid territories (Brdar-Jokanovic, 2020). The regions suffering from excess B accumulation were commonly reported for high B content in irrigation water and soil with little rainfall and insufficient leaching (Nable et al., 1997; Camacho-Cristóbal et al., 2018). B, which is taken up by plants in the form of boric acid, usually enters *via* passive diffusion in normal B conditions; however, boric acid channels and borate exporters are required for the movement of B within plants under B toxic environment (Pandey et al., 2019).

Though the role of B has been majorly recognized in the case of binding with the pectin polysaccharides present in the cell wall and thus facilitating cell wall stability (Funakawa and Miwa, 2015), its involvement in other metabolic processes has also been constantly highlighted (Hamurcu et al., 2020a). Thus, its excess leads to additional accumulation in apoplastic and symplastic tissues binding with molecules, including sugar molecules, glycoproteins, glycolipids, and ribose containing compounds such as RNA, ATP, and NADPH (Reid et al., 2004), and consequently hampering important processes such as cell division, photosynthesis, and stomatal conductance (Papadakis et al., 2004; Landi et al., 2012).

Along with the hindrance in root growth, the main symptoms of B toxicity include necrosis on leaf tips and chlorosis, which results from the production of reactive oxygen species (ROS) (Cervilla et al., 2007; Wang et al., 2010; Sakamoto et al., 2011). However, the reactions toward B toxicity rely on the capacity of the plant to re-translocate B within the phloem. Though it is thought that genotypes with less B uptake from roots - and consequently lower transport to shoots - can show greater tolerance to B toxicity, some genotypes with a greater B uptake and higher shoot B (SB) concentration can also be tolerant to high B (Reid and Fitzpatrick, 2009). Hence, B toxicity tolerance varies considerably within the species and among different species (Papadakis et al., 2003; Cervilla et al., 2007; Ardic et al., 2009; Landi et al., 2013; Wu et al., 2018). Thus, greater genetic diversity in the gene pool of a plant species is a desirable character that can help to deal with the problem of B toxicity stress.

Wheat is one of the main cereal crops and its yield is largely affected by high soil B either alone or in combination with salinity stress, i.e., BorSal stress (Pandey et al., 2019). A number of studies have reported a differential response of wheat genotypes toward B toxicity stress (Paull, 1990; Yau et al., 1997; Torun et al., 2006). However, these reported variations were dependent on several factors, including the growth stage (young or old plant), a dosage of B treatment, growth environment (hydroponic or soil), genotypes, and the organs in which B concentration is determined (shoot, root, or grains) (Brdar-Jokanovic, 2020). Thus, the B tolerance level of wheat genotypes may vary irrespective of the fact whether B concentration in different tissues is high or low (Yau et al., 1994).

Moreover, despite the attempts to find a correlation between B tolerance and geographical origins of genotypes (Yau et al., 1995; Rerkasem and Jamjod, 1997; Kalayci et al., 1998; Rerkasem et al., 2004; Pallotta et al., 2014; Brdar et al., 2017), no final conclusion could be drawn on the basis of origin (Brdar-Jokanovic, 2020). Though a variation in B toxicity tolerance has been observed in the existing cultivated wheat gene pool, the genetic base of the germplasm is still narrow and needs to be broadened for crop improvement (Rerkasem et al., 2004). To achieve the genotypes that may adjust to a high range of soil B concentration, it is necessary to identify B toxicity-tolerant sources from all the wheat gene pools and to include them in the breeding programs.

Wild wheat relatives have been identified as a potential source of genes providing tolerance to different biotic and abiotic stress conditions (Hairat and Khurana, 2015; Ahmadi et al., 2018; Nazari et al., 2018; Olivera et al., 2018). Aegilops, which is the closest genus to cultivated wheat, have been explored to a certain extent for B toxicity tolerance (Emon et al., 2012). While some of the Aegliops accessions, including *A. tauschii, A. longissima*, and *A. sharonensis*, have shown certain B tolerance, there is scanty information on the status of lipid peroxidation and proline formation in this tertiary wheat gene pool under high B stress.

**FIGURE 1:**
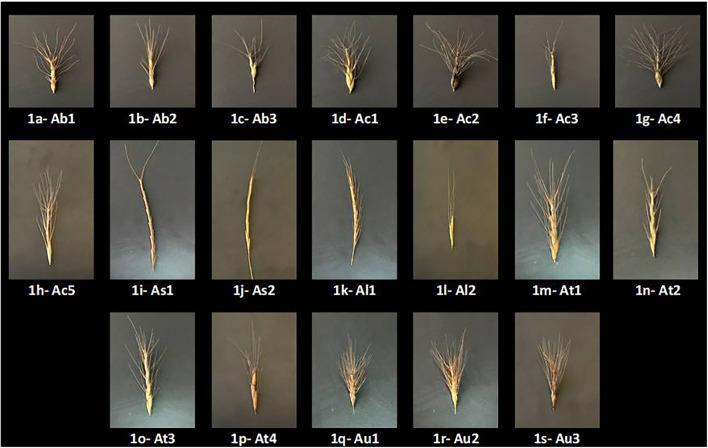
Pictures of the 19 Aegilops accessions provided by the Turkish Seed Gene Bank (TSGB), Ankara, and AARI National Gene Bank, İzmir, Turkey. **(a)** Ab1 (*Aegilops biuncialis*1: TGB 026218; 4x); **(b)** Ab2 (*A. biuncialis*2: TGB 026219; 4x); **(c)** Ab3 (*A. biuncialis3*: TGB 037313; 4x); **(d)** Ac1 (*A. columnaris1*: TGB 037373; 4x); **(e)** Ac2 (*A. columnaris2*: TGB 038488; 4x); **(f)** Ac3 (*A. columnaris3*: TGB 037489; 4x); **(g)** Ac4 (*A. columnaris4*: TGB 000107; 4x); **(h)** Ac5 (*A. columnaris5*: TR 57295; 4x); **(i)** As1 (*A. speltoides1*: TGB 037791; 2x); **(j)** As2 (*A. speltoides2*: TR 62174; 2x); **(k)** Al1 (*A. ligustica1*: TGB 000803; 2x); **(l)** Al2 (*A. ligustica2*: TR 39488; 2x); **(m)** At1 (*A. triuncialis1*: TGB 037311; 4x); **(n)** At2 (*A. triuncialis2*: TGB 037355; 4x); **(o)** At3 (*A. triuncialis3*: TGB 037376; 4x); **(p)** At4 (*A. triuncialis4*: TR 72224; 4x); **(q)** Au1 (*A. umbellulata1*: TGB 037353; 2x); **(r)** Au2 (*A. umbellulata2*: TGB 037356; 2x); **(s)** Au3 (*A. umbellulata1*: TR 72200; 2x).

Thus, we investigated the disparity in the responses of 19 different accessions (Figure 1) of 6 different Aegilops species toward a high B supply as compared to the well-recognized B toxicity-tolerant cultivar “Bolal 2973” (Mahboobi et al., 2001; Öz et al., 2014; Pallotta et al., 2014) in terms of root and shoot growth parameters, lipid peroxidation, proline content, and B accumulation. The aim of this study is to determine the level of resilience (most tolerant accession) in these genotypes toward B toxicity, which could contribute to an understanding of its adaptive mechanism under B stress conditions and for the improvement of high B tolerance in wheat breeding programs.

## Methodology

### Plant Materials

Aegilops genotypes used in this study consisted of 19 accessions belonging to 6 different species (3 diploid and 3 tetraploid species) (Table 1). The materials provided by the Turkish Seed Gene Bank (TSGB), Ankara, and AARI National Gene Bank, İzmir, Turkey were originated from the different sites of Turkey, and their respective gene banks accession numbers are listed in Table 1. To refer to a particular accession in the text, their assigned genotype code is used. The Turkish bread wheat cultivar, Bolal 2973, well recognized for its B toxicity tolerance (Mahboobi et al., 2001; Öz et al., 2014; Pallotta et al., 2014), was used as reference material in that study mainly to estimate the extent of damage to the root and shoot tissues in the presence of a high B supply.

**TABLE 1:** Abbreviation code, Genbank code, genome, ploidy, and the site of origin of the 19 Aegilops accessions along with the bread wheat cultivar, Bolal 2973 used in this study.

Abbreviation code	Genbank code	Taxon	Genome	Ploidy	Site of origin
Ab1	TGB 026218	*Aegilops biuncialis*	UUMM	4x	Adıyaman, Turkey
Ab2	TGB 026219	*Aegilops biuncialis*	UUMM	4x	Şanlıurfa, Turkey
Ab3	TGB 037313	*Aegilops biuncialis*	UUMM	4x	Gaziantep, Turkey
Ac1	TGB 037373	*Aegilops columnaris*	UUMM	4x	Gaziantep, Turkey
Ac2	TGB 038488	*Aegilops columnaris*	UUMM	4x	Ankara, Turkey
Ac3	TGB 037489	*Aegilops columnaris*	UUMM	4x	Şanlıurfa, Turkey
Ac4	TGB 000107	*Aegilops columnaris*	UUMM	4x	Adıyaman, Turkey
Ac5	TR 57295	*Aegilops columnaris*	UUMM	4x	Van, Turkey
As1	TGB 037791	*Aegilops speltoides*	SS	2x	Şanlıurfa, Turkey
As2	TR 62174	*Aegilops speltoides*	SS	2x	Gaziantep, Turkey
Al1	TGB 000803	*Aegilops ligustica*	SS	2x	Mersin, Turkey
Al2	TR 39488	*Aegilops ligustica*	SS	2x	Şanlıurfa, Turkey
At1	TGB 037311	*Aegilops triuncialis*	CCUU	4x	Şanlıurfa, Turkey
At2	TGB 037355	*Aegilops triuncialis*	CCUU	4x	Adıyaman, Turkey
At3	TGB 037376	*Aegilops triuncialis*	CCUU	4x	Gaziantep, Turkey
At4	TR 72224	*Aegilops triuncialis*	CCUU	4x	Adıyaman, Turkey
Au1	TGB 037353	*Aegilops umbellulata*	UU	2x	Erzincan, Turkey
Au2	TGB 037356	*Aegilops umbellulata*	UU	2x	Şanlıurfa, Turkey
Au3	TR 72200	*Aegilops umbellulata*	UU	2x	Şanlıurfa, Turkey
Bolal	Bolal 2973	*Triticum aestivum*	AABBDD	6x	Turkey

### Experimental Conditions and Measurement of Growth Parameters

For evaluating the effect of B toxicity stress on the growth of Aegilops accessions, the experiment was conducted in the hydroponic experiment that was adjusted to 45–55% humidity, 16/8 h light/dark photoperiod, 22 ± 10°C temperature, and 16,000 Lx/day light intensity. All the accessions along with the check cultivar were grown three times in three different treatments [Control (1/5th Hoagland solution containing 3.1 μM B), toxic B (1 mM B), and highly toxic B (10 mM B)] following a randomized design. Seeds used in this experiment were surface sterilized using sodium hypochlorite, 75% ethanol, and water and were further kept in darkness at 22°C for 3 days for germination.

After germination, five seedlings of all the genotypes were transferred to sterile hydroponic pots (containing one-fifth of Hoagland’s solution) three times in three different sets (one set for each B treatment). Thus, 1 biological replicate comprised 5 plants, and a total of 15 plants per accession were kept for each B treatment. After 3 days of growth, plants were supplied with Hoagland’s solution (Control) and required B concentrations, 1 mM B and 10 mM B. B treatment was given for 7 days, and the nutrient solution was changed after every 3 days.

The root and shoot harvest was done on the 7th day of B treatment when the plants were in the tillering stage (Feekes scale 4–5) (Supplementary Figure 1), and the samples for the different analyses were collected in triplicates. Immediately after harvest, the root lengths (RLs) and shoot lengths (SLs) of the plants were measured, and root fresh weight (RFW) and shoot fresh weight (SFW) was weighed (Supplementary Figures 2A,B). Further, the root and shoot samples of the plants were kept in an oven at 70°C for 72 h, and further, root dry weights (RDWs) and shoot dry weights (SDWs) were estimated. Additionally, the root and shoot samples were collected for malondialdehyde (MDA), proline, and inductively coupled plasma atomic emission spectrometry (ICP-AES) analysis.

### Inductively Coupled Plasma-Atomic Emission Spectrometry Analysis for the Estimation of Root-Shoot B Content

For the ICP-AES analysis, the harvested root and shoot samples were washed with 0.1 N HCl solution and deionized water followed by air-drying of leaf samples at 70°C in a hot air oven. Dried samples were crushed, and 0.15–0.20 g of the powdered sample was dissolved in 5 ml of 65% HNO3 and 2 ml of 35% H2O2 and digested in a closed microwave accelerating reaction system (Cem Marsxpress, Matthews, NC, United States). ICP-AES (Varian, Vista, Palo Alto, CA, United States) was used to determine the nutrient concentration in the stock solution (Pandey et al., 2016). The measurement of the elemental concentration was checked by the certified values of a B element in the reference leaf material provided by the National Institute of Standards and Technology (NIST, Gaithersburg, MD, United States).

### Lipid Peroxide Estimation

Malondialdehyde is an end product of lipid peroxidation. Thus, a low MDA content shows less lipid peroxidation and consequently less damage in plant tissues under a stress condition. Reduced damage to plant tissues indicates a greater tolerance level of plants (Esim et al., 2013). Hence, the MDA level in the plant is considered a good indicator of plant tolerance to a particular stress condition. Thus, in this study, its level was estimated by employing the thiobarbituric acid method (Rao and Sresty, 2000; Hamurcu et al., 2020a). For MDA estimation, firstly, 0.5 g of the root and shoot samples were homogenized in 2.5 ml of trichloroacetic acid (TCA) (0.1%) followed by centrifugation at 10,000 × *g* for 10 min. After centrifugation, 1 ml supernatant was dissolved with 4 ml of 20% TCA containing 0.5% thiobarbituric acid. The mixture was further heated to 30 min at 95°C and then kept in an ice bath for cooling followed by centrifugation at 10,000 *g* for 15 min. The absorbance of supernatants was estimated at 532 nm, and the final concentration was calculated using an extinction coefficient of 155 mM^–1^ cm^–1^.

### Proline Level Estimation

In many plants, a strong correlation has been established between proline accumulation and abiotic stress tolerance (Hare and Cress, 1997; Kishor et al., 2005; Kavii Kishor and Sreenivasulu, 2014). Thus, it is interesting to observe the level of proline accumulation in tolerant genotypes under toxic B stressed conditions to understand the role of proline content in providing B toxicity tolerance. Thus, for the estimation of the proline content of the treated root and shoot samples, firstly, the acid ninhydrin was freshly prepared by heating 1.25 g ninhydrin in 30 ml glacial acetic acid and 20 ml 6 M phosphoric acid and stored at 4°C for 24 h (Bates et al., 1973; Hamurcu et al., 2020b). Further, about 0.5 g of the sample was homogenized in 3% sulphosalicylic acid and filtered. Afterward, 2 ml of each, acid ninhydrin and glacial acetic acid was added to 2 ml of extracted filtrate and kept at 100°C for 1 h. Further, the reaction was stopped by keeping the sample in an ice bath. The reaction mixture was mixed with 4 ml toluene using a stirrer for 15–20 s. Finally, the absorbance of the aspired part containing toluene was measured at 520 nm.

### Data Analysis

To identify the accessions that are tolerant to B toxicity, statistical analysis was performed on the data from the measurements of different traits. The percentage changes in each of the measured physiological traits under 1 mM and 10 mM B treatment as compared to Control (based on three replicates) were estimated using MS Excel 2010. The frequency distribution analysis based on the percentage changes under 10 mM B treatment as compared to Control for all the measured traits was performed using Minitab Statistical software version 16. The normal graph was plotted for all the measured traits (Supplementary Figure 3). The obtained data were subjected to two-way ANOVA using the Graphpad prism 9.0 program, where treatments and genotypes were the two factors and the trait observed was the response. The role of genotypes (G), treatments (T), and their (G × T) interaction in the variability in the expression of traits was considered to be highly significant for the values with *p* < 0.001. The mean differences between the two B treatments as compared to Control were calculated for the different traits using Dunnett’s multiple comparisons tests, and the comparisons with *p* < 0.001 were considered to be significantly different. Bar diagrams based on the average of the three replicates with SE mean caps were developed to estimate the extent of the responsiveness of Aegilops accessions under B toxicity stress. Tukey’s pairwise comparison using the general linear model of Minitab Statistical software version 16 was employed as a *post hoc* analysis to distinguish any significant differences among the experimental genotypes. A correlation analysis employing a Pearson correlation was conducted using the Minitab v. 16 software to find an association between the relative changes of the studied parameters, and the associations with *p* < 0.05 were considered to be significant. A heatmap employing the average linkage method was generated using the Euclidean distance matrix based on the percentage changes in the trait values. It allowed the clustering of all the experimental genotypes based on their responses toward B toxicity stress and the grouping of the studied traits according to their association with each other.

## Results

A set of 19 accessions consisting of diploid (*Aegilops speltoides*, *Aegilops ligustica*, and *Aegilops umbellulata*) and tetraploid (*Aegilops biuncialis, Aegilops columnaris*, and *Aegilops triuncialis*) progenitor species along with the Control wheat cultivar, Bolal 2973, were subjected to two different B toxicity stress conditions to evaluate their responses (Table 1). The hydroponic experiment was conducted to observe the changes in growth parameters, B accumulation, MDA, and proline content in the roots and shoots of the treated plants in response to B toxicity stress. The overall aim of this study is to determine a variation in the resilience of these Aegilops accessions under toxic B growth conditions.

### ANOVA for All the Studied Traits

ANOVA-based assessment of the effect of B toxicity stress in 19 Aegilops accessions revealed a significant genotypic effect for all the studied traits, except RB and SB accumulation (Table 2). These significant variations among the accessions specify a high genetic diversity in the studied accessions. Indeed, the experimental accessions could serve as an appropriate gene pool for the B toxicity tolerance-based breeding programs. B toxicity stress treatment was also contributed significantly as a source of variation for all the studied traits (Table 2). However, Dunnett’s test revealed that among all the studied traits, the mean difference between Control vs. 1 mM B treatment was significant only for the root malondialdehyde (RMDA) and shoot malondialdehyde (SMDA) and proline content. However, the mean difference between Control vs. 10 mM B treatment was significant for all the studied traits (Table 2). Thus, a variation in the measured traits under 10 mM B treatment is mainly focused on while discussing the results of this study.

**TABLE 2:** Details of the results obtained from the two-way ANOVA showing the impact of genotypes (G), B toxicity treatments (T), and their interaction (G × T) on individually studied traits representative of plant growth and development.

Studied traits	Code	% of total variation			*P* value			Control vs. 1 mM		Control vs. 10 mM	
		Interaction	Genotypes	Treatment	Interaction	Genotypes	Treatment	Mean difference	Adjusted *P* value	Mean difference	Adjusted *P* value
Root Length	RL	13.1	50.5	22.8	****	****	****	–0.117	ns	8.833	****
Shoot Length	SL	14.7	54.5	4.3	*	****	***	0.450	ns	2.050	****
Root Fresh Weight	RFW	12.4	30.0	23.2	ns	****	****	0.009	ns	0.084	****
Shoot Fresh Weight	SFW	6.9	57.0	15.4	ns	****	****	0.006	ns	0.087	****
Root Dry Weight	RDW	10.3	45.4	19.6	ns	****	****	0.001	ns	0.004	****
Shoot Dry Weight	SDW	9.0	64.8	2.4	ns	****	**	0.000	ns	0.004	**
Root MDA Content	RMDA	17.2	43.0	39.2	****	****	****	–2.132	****	–4.806	****
Shoot MDA Content	SMDA	18.4	41.0	40.5	****	****	****	–7.194	****	–13.290	****
Root Proline Content	Rpro	19.3	57.9	20.6	****	****	****	–0.013	****	–0.020	****
Shoot Proline Content	Spro	19.9	59.7	18.8	****	****	****	–0.013	****	–0.021	****
Root B Accumulation	RB	−	27.1	31.1	-	ns	****	–0.728	ns	–4.649	****
Shoot B Accumulation	SB	−	15.0	58.7	-	ns	****	–13.790	ns	–88.590	****

Significant differences are indicated as **** for *p* < 0.0001; *** for *p* < 0.0005; ** for *p* < 0.005; * for *p* < 0.05; ns, non-significant.

### Impact of B Toxicity Stress on the Root-Shoot Length

The average RL of Aegilops accessions in the Control group ranged from 9.50 to 35.67 cm, whereas it was from 5.50 to 19.67 cm in 10 mM B treatment (Supplementary Table 1). A highly toxic B led to a reduction in RL up to 133% in Aegilops species (shown by the accession At3) and a decrease of 49% of Bolal 2973 as compared to the Control treatment. However, a number of Aegilops accessions, including Ab2, Ac4, As2, Al1, and At2, showed less reduction in RL as compared to the check cultivar, Bolal 2973 under a highly toxic B condition. Among these accessions, Ab2 and Al1 showed the lowest reduction of 14% and 10% in RL, respectively (Table 3). In the case of RL, ANOVA showed a significant difference in genotypes (G), B toxicity treatment (T), and their interaction (G × T) (Table 2).

**TABLE 3:** The percentage change in the studied traits of 19 Aegilops accessions and the B-tolerant check cultivar, Bolal 2973 is highly toxic B (10 mM B) treatment as compared to Control.

Code	SL	RL	SFW	RFW	SDW	RDW	S_MDA	R_MDA	S_Proline	R_Proline	S_B	R_B
Ab1	–1.6	–42.5	–21.6	–92.3	6.1	–28.0	47.3	33.2	28.6	33.2	99.26	98.08
Ab2	11.9	–14.0	–8.5	–47.1	30.6	–8.7	33.3	31.1	46.9	51.7	99.61	98.18
Ab3	–1.4	–87.9	–35.1	–114.3	–0.3	–47.2	61.2	21.0	31.3	40.6	99.50	99.36
Ac1	–16.1	–117.3	–48.2	–185.0	–6.6	–91.6	51.3	50.4	–5.4	55.7	99.20	90.11
Ac2	–20.8	–65.9	–98.0	–175.0	–20.2	–75.9	56.2	45.7	25.0	54.4	99.58	97.29
Ac3	–19.5	–117.2	–89.5	–403.1	–47.9	–192.3	46.6	31.1	77.6	41.7	99.17	93.70
Ac4	12.0	–31.8	–4.6	–13.6	20.0	–13.0	47.3	13.6	61.1	43.5	99.81	99.08
Ac5	–9.0	–81.6	–95.5	–109.0	–29.7	–79.1	49.2	43.4	57.0	23.2	98.64	91.35
As1	–6.5	–68.5	–125.3	–356.0	–81.9	–206.1	45.7	25.6	31.6	42.8	99.43	94.02
As2	–2.0	–19.5	–66.2	–134.6	–27.0	–89.8	60.0	31.8	68.3	57.7	99.03	90.12
Al1	6.8	–10.5	–36.5	–100.0	–18.7	–20.0	13.9	36.8	16.9	47.0	98.95	96.66
Al2	–23.7	–67.0	–187.9	–404.4	–98.4	–193.5	60.9	15.8	72.8	7.2	98.91	86.93
At1	–24.1	–65.9	–80.2	–204.4	–53.8	–131.4	62.8	63.4	54.4	48.1	99.09	97.48
At2	–19.4	–40.6	–30.7	–83.4	–4.0	–28.1	47.3	24.3	36.7	45.5	99.02	94.73
At3	–20.8	–133.8	–109.4	–447.8	–70.7	–148.5	53.0	57.8	65.8	69.4	98.94	96.28
At4	–0.7	–81.4	–33.0	–192.2	–27.5	–174.8	50.3	56.2	19.7	29.0	99.15	96.20
Au1	–9.6	–102.6	–51.5	–178.7	–5.6	–49.1	41.4	46.1	46.3	55.9	99.56	96.07
Au2	0.8	–73.4	–50.1	–262.8	–20.0	–148.1	49.4	60.6	14.0	42.5	99.72	97.99
Au3	–13.1	–90.0	–67.2	–232.5	–19.0	–95.7	42.3	49.7	–89.8	–26.1	99.46	92.57
Bolal	–38.2	–49.5	–30.0	–108.4	–0.3	–52.6	33.2	8.9	68.0	68.4	99.09	91.34

The SL of Aegilops accessions varied from 18.66 to 27.00 cm under controlled conditions, whereas it ranged from 16.50 to 23.67 cm under highly toxic B conditions (Supplementary Table 1). B toxicity developed due to boric acid led to a 24% reduction in SL in Aegilops species, while the same led to a 38% reduction in the SL of the B-tolerant check cultivar, Bolal 2973. Under highly toxic B stress, a substantial increase of around 11.9% was observed in the SL of Ab2 and Ac4. Ab2 accession also showed the highest increase in RL (Table 3). ANOVA showed a significant difference among genotypes (G), B toxicity treatment (T), and their interaction (G × T) in SL (Table 2).

### Impact of B Toxicity Stress on the Root-Shoot Fresh Weight

Under controlled growth conditions, the highest RFW was observed in Au2 (270 mg) followed by Ac1 (246 mg), which was higher than the B-tolerant cultivar Bolal (140 mg) (Supplementary Table 1). However, under a highly toxic B condition, Ac1 showed the highest RFW followed by Ac5, Au2, and Ac4. A minimum reduction (13%) in RFW was observed in Ac4 followed by Ab2 (47%) (Table 3). In RFW, the differences in both genotypes and treatment were found to be significant while the interaction (G × T) was found to be insignificant (Table 2).

The SFW of Aegilops species ranged from 100 to 394 mg under normal growth conditions, whereas under a highly toxic B supply, it varied between 65 and 266 mg (Supplementary Table 1). Though the check cultivar, Bolal showed the highest SFW under 10 mM B treatment, it showed a 30% reduction as compared to the Control treatment. Three Aegilops accessions, Ab1, Ab2, and Ac4, showed a lower reduction in SFW as compared to Bolal. The lowest reduction of around 4% has been observed in Ac4 (Table 3). The interaction between the genotypes and stress treatment was not significant in the case of SFW; however, both B toxicity treatment and genotypes individually revealed significant differences (Table 2).

### Impact of B Toxicity Stress on the Root-Shoot Dry Weight

Root dry weight and shoot dry weight under B toxic growth conditions have been widely considered for the understanding of the level of B tolerance in wheat genotypes (Torun et al., 2006; Metwally et al., 2012; Nejad et al., 2015). Aegilops accessions showed wide variability in RDW under controlled growth conditions ranging from 3 to 15 mg and a highly toxic B growth condition ranging from 2 to 8 mg (Supplementary Table 1). The check cultivar Bolal 2973 showed a 52% decrease with 11 mg RDW under normal growth conditions and 7 mg RDW under 10 mM B treatment. Though seven accessions showed less decrease in RDW as compared to the check cultivar, the accessions Ab2 (9%) and Ac4 (13%) showed the lowest decrease (Table 3). According to the obtained ANOVA results, both genotypes and treatment significantly act as a source of variation in the case of RDW. However, the interaction of the two was not significant (Table 2).

The SDW of B-tolerant cultivar, Bolal remains almost the same both under controlled growth and highly toxic B conditions (53 mg) (Supplementary Table 1). However, the three Aegilops accessions, Ab1, Ab2, and Ac4, showed a 6, 31, and 20% increase in SDW under a highly stressed condition, respectively (Table 3). The difference in the treatment was significant (<0.01), whereas the difference of genotypes was highly significant (<0.0001) in the case of SDW. The interaction G × T was not significant (Table 2).

### Impact of B Toxicity Stress on the Root-Shoot Malondialdehyde Content

According to the obtained results, the range of MDA content in roots is found to be less than the shoots. While RMDA content varied from 5.60 to 12.61 nmol g^–1^ FW under the Control treatment, it changed from 8.04 to 16.78 nmol g^–1^ FW under 10 mM B treatment (Supplementary Table 2). Among Aegilops accessions, Ac4 showed the least increment of only 13% under a highly toxic B environment as compared to Control, whereas the check cultivar, Bolal 2973 showed an increase of only 9% as compared to control (Table 3).

The SMDA content of Aegilops accessions varied from 7.28 to 21.55 nmol g^–1^ FW under a normal growth condition, whereas the same was in the range between 15.60 and 49.18 nmol g^–1^ FW under highly toxic B treatment (Supplementary Table 2). In highly toxic B supply, though most of the genotypes showed an increase in SMDA content as compared to the check cultivar Bolal, the increment was least in the accession, Al1. Among Aegilops accessions, Ac1 and Ac2 showed the lowest (15.6 nmol g^–1^ FW) and highest (49.1 nmol g^–1^ FW) SMDA content under a highly toxic B environment (Table 3). In the case of MDA, all of the three [genotypes (G), treatment (T), and their interaction (G × T)] acted as a significant source of variation in both roots and shoots (Table 2).

### Impact of B Toxicity Stress on the Root-Shoot Proline Content

The root proline (RProline) content of Aegilops accessions ranged from 0.013 to 0.060 nmol g^–1^ FW and 0.024 to 0.085 nmol g^–1^ FW under Control and 10 mM B treatment, respectively (Supplementary Table 2). With a decrease of 26% and an increase of only 7%, the Aegilops accessions, Au3 and Al2, respectively, showed the least increment in RProline content. The Aegilops accession, At3 (69%), and the check cultivar, Bolal 2973 (68%), showed a maximum increase in RProline content under 10 mM B treatment as compared to Control (Table 3).

A huge variation in the shoot proline (SProline) content of Aegilops accessions was observed under both Control (0.009–0.063 nmol g^–1^ FW) and 10 mM B treatment (0.011–0.089 nmol g^–1^ FW). While Au3 showed an 89% decrement in the proline content under a highly toxic B condition as compared to Control, Ac3 revealed the highest increase (78%) in proline content as compared to Control (Table 3).

Similar to MDA, in the case of proline also, all of the three (genotypes, treatment, and their interaction) acted as a significant source of variation in both roots and shoots. However, the genotypes showed a much greater contribution to the variability as compared to treatments (Table 2).

### Impact of B Toxicity Stress on the Root-Shoot B Accumulation

In root B (RB) accumulation, Ab3 and Ac4 showed a maximum increase of 99.36 and 99.08% under highly toxic B treatment as compared to Control (Table 3). The check cultivar Bolal showed a 91.34% increase in B accumulation under 10 mM B treatment as compared to Control. In 10 mM B treatment, Ab1 (16.97 μg^–1^ DW) and Ab3 (17.80 μg^–1^ DW) showed the maximum, and Al1 (0.75 μg g^–1^ DW) and Al2 (0.98 μg g^–1^ DW) demonstrated minimum RB accumulation (Supplementary Table 2).

The accumulated B content in the shoot of Aegilops accessions ranged from 0.28 to 0.81 μg g^–1^ DW under the Control treatment, whereas the same varied from 30.52 to 295.62 μg g^–1^ DW under highly toxic B treatment (Supplementary Table 2). While Ac4 showed a maximum increase in B accumulation (99.81%) as compared to Control, Ac5 revealed a minimum increase (98.64%) (Table 3). As2 and Al1 showed a minimum SB accumulation of 30.52 and 42.65 μg g^–1^ DW (Supplementary Table 2). In the case of RB and SB content, differences in the treatment were found to be significant, which contribute to a 59% variation in shoots and a 31% variation in roots whereas the differences in the genotypes were found to be non-significant (Table 2).

### Correlation Between the Measured Traits of Aegilops Accessions Grown Under B Toxic Condition

The association between the relative changes in the different studied traits was estimated using a Pearson correlation. The analysis revealed significant positive correlations between the biomass (SDW and RDW) and the growth parameters (SL and RL) (Table 4). The RProline and SProline content were not significantly associated with any of the growth parameters or MDA content; however, a significant positive correlation was found between the RProline and SProline content. The RMDA and SMDA content were mostly negatively correlated with all the growth parameters; however, these correlations were not significant except SL. Interestingly, RB showed a significant positive correlation with all the shoot-based traits, including SL, SFW, SDW, and SB. This directs toward the role of B uptake from the roots in SB accumulation and consequently affecting the shoot growth in terms of length and dry weight. SB was found to be significantly and positively associated with SDW only. Other than this, no significant correlation was observed between SB and the measured traits (Table 4).

**TABLE 4:** Pearson’s correlation and its significance summarizing the association between the percentage changes in the studied parameters under highly toxic B (10 mM B) treatment as compared to Control.

	SMDA	RMDA	SPro	RPro	SL	RL	SFW	RFW	SDW	RDW	SB
RMDA	0.05										
*p*-value	0.837										
SPro	0.27	–0.33									
*p*-value	0.265	0.169									
RPro	0.01	0.10	0.56*								
*p*-value	0.967	0.698	0.013								
SL	−0.51*	–0.27	–0.05	0.08							
*p*-value	0.026	0.266	0.825	0.739							
RL	–0.35	–0.44	0.10	0.04	0.59**						
*p*-value	0.138	0.063	0.684	0.887	0.008						
SFW	–0.40	0.01	–0.23	0.21	0.68**	0.37					
*p*-value	0.094	0.973	0.335	0.379	0.002	0.115					
RFW	–0.27	–0.22	–0.12	0.09	0.61**	0.65	0.78**				
*p*-value	0.256	0.375	0.612	0.727	0.006	0.003	0.000				
SDW	–0.34	–0.10	–0.25	0.14	0.62**	0.38	0.91**	0.86**			
*p*-value	0.153	0.677	0.306	0.575	0.005	0.107	0.000	0.000			
RDW	–0.40	–0.25	–0.12	0.17	0.49*	0.53*	0.72**	0.88**	0.85**		
*p*-value	0.091	0.304	0.623	0.484	0.033	0.020	0.000	0.000	0.000		
SB	–0.071	–0.092	–0.273	0.065	0.427	0.096	0.421	0.229	0.492*	0.234	
*p*-value	0.773	0.708	0.257	0.792	0.068	0.695	0.073	0.345	0.032	0.336	
RB	–0.22	0.12	–0.01	0.35	0.46*	0.19	0.63**	0.39	0.52*	0.42	0.57*
*p*-value	0.365	0.618	0.965	0.143	0.049	0.439	0.004	0.100	0.023	0.072	0.011

Associations with *p* < 0.05 and *p* < 0.01 were considered to be significant (*) and highly significant (**), respectively.

### B Toxicity-Tolerant Aegilops Accessions

**FIGURE 2:**
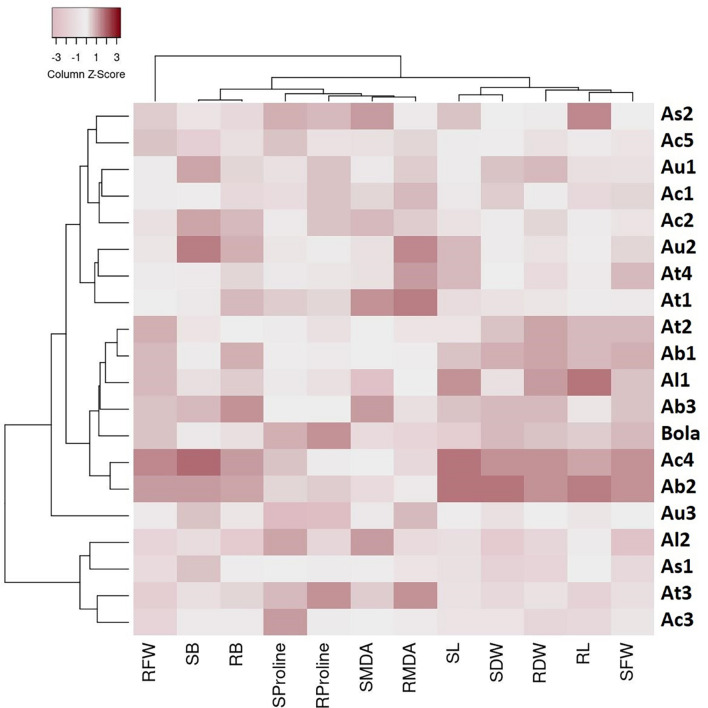
A heat map describing the boron (B) toxicity stress-induced genetic variability in traits among 19 Aegilops accessions and the check cultivar, Bolal 2973 grown under B toxicity in the hydroponic system. The color code signifies the *Z* score for each genotype: dark color specifies an increase in the percentage of the trait values of the genotypes under B toxicity as compared to Control, whereas a lighter shade specifies the decrease in the percentage of the trait values of the genotypes under B toxicity as compared to Control. The genotypes were clustered into three different groups (C1–C3) based on their response to B toxicity stress in terms of different traits, including root length (RL), shoot length (SL), RFW (root fresh weight), shoot fresh weight (SFW), root dry weight (RDW), shoot dry weight (SDW), root malondialdehyde (RMDA), shoot malondialdehyde (SMDA), root proline (RProline), shoot proline (SProline), root boron (RB accumulation), and shoot boron (SB accumulation).

The cluster analysis-based heat map presented in Figure 2 depicts the physiological responses of 19 Aegilops accessions toward B toxicity stress. Three main clusters with distinguished genetic profiles were identified based on the percentage changes in the studied traits in response to a high B supply. Cluster 1 comprises six Aegilops accessions and one B-tolerant check cultivar, Bolal. The accessions in Cluster 1 had mostly higher SL, RL, SFW, RFW, SDW, and RDW in response to highly toxic B stress in comparison with Clusters 2 and 3 and thus classified as tolerant genotypes. In this cluster, two genotypes, Ab2 and Ac4, were categorized as the most tolerant genotypes as they showed a maximum increment or least decrement in all the growth parameters, including RL and SL and biomass production (RDW and SDW). The eight genotypes in Cluster 2 were considered as moderate genotypes based on their responses toward high B. These accessions mostly showed lower SL, RL, SFW, RFW, SDW, and RDW in response to highly toxic B stress as compared to the accessions in Cluster 1 but higher values than the accessions in Cluster 3. The third group comprises four accessions, which showed lower SL, RL, SFW, RFW, SDW, and RDW in response to highly toxic B stress as compared to both Clusters 1 and 2. Moreover, the heat map also grouped the studied traits into two clusters, where most of the growth parameters, including SL, RL, SFW, SDW, and RDW, are clustered together. In addition, SB and RB were grouped together. These groupings were consistent with the results obtained in Pearson correlation analysis, and the traits showing a significant association were clustered together in a heat map as well.

It can be observed from the heat map (Figure 2) that Cluster 1 contains the most tolerant accessions, comprising the genotypes from all the species, including *A. biuncialis, A. triuncialis, A. columnaris, A. ligustica*, and Bolal. However, the other genotypes of the same species were also present in Clusters 2 and 3 that did not show symptoms of B toxicity tolerance. Thus, it cannot be concluded that high tolerance was specific to any of the species. Similarly, although most of the genotypes in the tolerant Cluster 1 were tetraploid, one of the genotypes, Al1 was diploid. Moreover, Clusters 2 and 3 are also comprised the genotypes with all the ploidy levels. Thus, it will not be appropriate to conclude that the high tolerance characteristics were related to the ploidy level. Consequently, it can be concluded that B toxicity tolerance in the experiment was not correlated to any ploidy or species but was genotype-dependent.

## Discussion

The process of domestication has narrowed the genetic variability of the genes responsible for the different valuable traits in wheat. Aegilops, the closest wheat relative, can serve as an important source of novel alleles, which can contribute to increasing this genetic diversity in wheat. Although most of the Aegilops species belong to the tertiary gene pool of wheat and thus face the challenges of incompatibility and crossability, a number of Aegilops genes have been utilized for wheat improvement in terms of biotic/abiotic stresses and nutritional development.

Aegilops have been extensively identified or utilized for different biotic stress conditions, including powdery mildew resistance (Tang et al., 2018), cereal cyst nematodes (CCN) resistance (Ali et al., 2019), leaf rust (Millet et al., 2014; Lee et al., 2020; Rani et al., 2020), stem rust (Yu et al., 2015; Edae et al., 2016; Huang et al., 2018; Olivera et al., 2018), stripe rust (Millet et al., 2014), green bug (Crespo-Herrera et al., 2013), and hessian fly (Martin-Sanchez et al., 2003; Kishii, 2019), and fusarium head blight (Brisco et al., 2017). Other than biotic stress, the utility of Aegilops species for abiotic stress, including drought (Djanaguiraman et al., 2019; Suneja et al., 2019), heat (Awlachew et al., 2016; Green et al., 2018), salinity stress (Kiani et al., 2015), has also been determined. Aegilops genes related to not only stress conditions but also to the grain protein content and grain quality of wheat have been reported (Neelam et al., 2012; Ma et al., 2013; Zhou et al., 2014; Garg et al., 2016; Du and Zhang, 2017; Rakszegi et al., 2017).

The effect of B toxicity stress on 19 Aegilops accessions from 6 different species to understand their adaptive mechanism and to identify the most tolerant accessions to B toxicity stress. The aim of this study was to identify the degree of tolerance in the screened accessions and to determine whether appropriate Aegilops accessions could be found and can be employed as genetic sources to improve B toxicity tolerance in cultivated wheat.

### Impact of B Toxicity Stress on the Root-Shoot Growth Parameters

**FIGURE 3:**
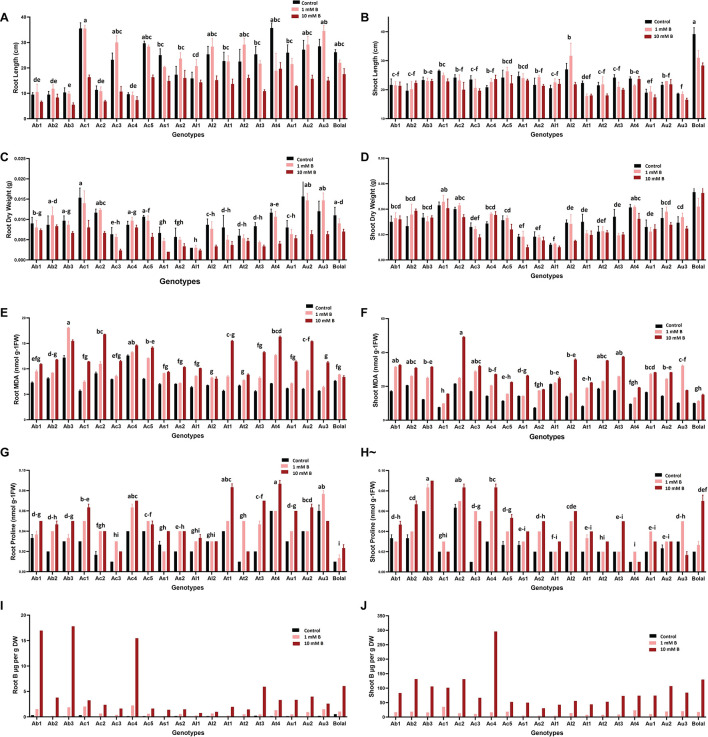
Variability in the **(A)** RL **(B)** SL **(C)** RDW **(D)** SDW **(E)** RMDA **(F)** SMDA **(G)** RProline **(H)** SProline **(I)** RB accumulation and **(J)** SB accumulation of 19 Aegilops accessions and the B-tolerant check cultivar, Bolal 2973 grown under Control (3.1 μM B), toxic B (1 mM B), and highly toxic B (10 mM B). Data represent means ± SE. Tukey’s pairwise comparison using the general linear model was employed to distinguish any significant differences among the experimental genotypes. Genotypes that do not share a letter are significantly different.

A number of studies have considered RL as the criteria for B toxicity tolerance (Schnurbusch et al., 2008; Pallotta et al., 2014). Genotypes showing an increment or a less decrement in RL under B toxic growth conditions as compared to Control can be selected as B tolerant (Figure 3A). In this study, the noticeable least decrement in terms of root development (RL and RDW) (Figures 3A,C) and a clear increment in terms of shoot development (SL and SDW) have been observed in some of the Aegilops accessions under highly toxic B growth conditions as compared to Control (Figures 3B,D). Ab2, Ac4, As2, Al1, and At2 showed less reduction in RL as compared to the check cultivar, Bolal. Similar to our study, Pallotta et al. (2014) found less reduction in the RL of the B-tolerant cultivar, Halbred under B toxic conditions as compared to the intolerant genotype, Cranbrook and Langdon.

Similar to RL, B toxicity may also negatively affect SL. However, in our study, despite having a general detrimental effect on the SL of the studied accessions, a very few accessions (Ab2, Ac4, and Al1) could tolerate the highly toxic B supply and showed a significant increase in SL (Figure 3B). Though a few studies reported no effect of B toxicity on SL (Yau and Saxena, 1997; Brdar-Jokanovic, 2020), others reported a significant negative effect of high B on the SL of wheat cultivars (Coskun et al., 2014). These differences in the results of the RL and SL direct toward a major role of genotypic variation in response to B toxicity. Thus, the accessions with less or no detrimental effects of high B on both RL and SL should be definitely considered for further studies to understand the mechanism behind B toxicity tolerance and for the B toxicity improvement in breeding programs.

Dry matter production, especially shoots under B toxicity, has also been accepted as a basis for the selection of the tolerant wheat genotypes (Torun et al., 2006; Metwally et al., 2012). In this study, Ab1, Ab2, and Ac4 showed an increment in SDW under a high B supply as compared to Control (Figure 3D). These results were consistent with the findings of Torun et al. (2006) where the genotypes with an increase in SDW were selected as tolerant. In the case of RDW, Ab2 and Ac4 showed less decrement under high B toxicity as compared to the check cultivar, Bolal (Figure 3C). It was similar to the results obtained by Nejad et al. (2015) where the varieties with a higher RDW than other varieties under the B toxic condition were accepted as tolerant. The damaging effect of B in our study was greater on RDWs as compared to the SDWs (20 and 2%, respectively). Similar observations were recorded by Kalayci et al. (1998) on wheat cultivars.

### Impact of B Toxicity Stress on the Root-Shoot Malondialdehyde Content

Malondialdehyde, a product of lipid peroxidation, has been consistently used as a marker to understand the extent of oxidative damage in plants due to abiotic stresses (Davey et al., 2005; Morales and Munné-Bosch, 2019). An increase in the RMDA and SMDA content in our study under a highly toxic B supply was consistent with a number of studies (Karabal et al., 2003; Esim et al., 2013; Mohamed et al., 2016; Choudhary et al., 2020). The percentage increase in the RMDA and SMDA content of Aegilops accessions was up to 63% under highly toxic B treatment as compared to Control (Table 3).

In roots, the lowest percentage increase in MDA content (9%) was observed in the tolerant check cultivar, Bolal showing the lowest oxidative damage in its roots. This might be responsible for a reduced decrease in its RL and dry weight under high toxic B in comparison with a maximum decrement obtained in the Aegilops accessions. Among Aegilops, the least increase (13%) in Ac4 accession reveals less lipid peroxidation in its roots (Figure 3E). As lipid peroxidation could be responsible for cell damage, a reduced increase of it seems to be accountable for the less damage and least decrement in its RDW.

In shoots, more than 30% increase has been observed in the MDA content of all the Aegilops accessions (except Al1, 13.5%) under 10 mM B treatment, which shows a significant level of lipid peroxidation due to high B toxicity (Figure 3F). However, Ab2 accession showed a 33% increase in SMDA content under highly toxic B, which was similar to the check cultivar Bolal. Similarly, an increase in the MDA content of Ac4 accession under a high B supply was also not too much (47%) as compared to the maximum increase (Table 3). This shows lesser oxidative damage in the shoots of these two accessions, and this could be a reason for an increase in their SL and dry weight in 10 mM B treatment.

### Impact of B Toxicity Stress on the Root-Shoot Proline Content

Proline is an osmoprotectant known for its role in plants in protecting the proteins from denaturation, the detoxification of hydroxyl radicals, and the stabilization of phospholipids especially under abiotic stress conditions (Cervilla et al., 2012). However, the effect of B toxicity on its accumulation in plants is still controversial (Pandey et al., 2019). While a few studies reported a significant increase in proline content under B toxic condition (Eraslan et al., 2007a; Kaya et al., 2018; Samet and Çıkılı, 2019), others discussed insignificant changes in its accumulation under B toxicity (Karabal et al., 2003; Molassiotis et al., 2006; Eraslan et al., 2007b).

In this study, the effect of B toxicity on proline accumulation and also its role in regulating lipid peroxidation is not conclusive. In general, the proline accumulation increased in both roots and shoots of the studied accessions under highly toxic B treatment. However, some of the accessions showed a decrease in the root (Au3) and shoot (Ac1 and Au3) proline content under a highly toxic B supply (Figures 3G,H).

It has been considered in several studies that under B toxic conditions, proline forms a complex with ROS and detoxifies their function with a detrimental effect on lipid peroxidation (Hong et al., 2000). Thus, a decrease in proline accumulation may lead to a higher extent of lipid peroxidation increasing the MDA content in the stressed tissues (Molassiotis et al., 2006). In contrary to this argument, in our study, the accessions with a maximum decrement in proline content did not show a maximum increase in MDA content. Thus, an association between proline accumulation and MDA content could not be concluded, which was in line with the findings of Karabal et al. (2003).

Interestingly, when the RProline and SProline content of the two Aegilops accessions, Ab2 and Ac4 (which demonstrated tolerance in root and shoot growth parameters), were observed under a high B supply, both accessions showed a proline content closer to a higher end in the range (although it was not the highest) (Supplementary Table 2). This directs toward the controlled oxidative tissue damage in them and further demonstrate either positive or less detrimental effect on their RL and SL and dry weights.

### Impact of B Toxicity Stress on the Root-Shoot B Accumulation

Variability in RB and SB accumulation in the studied Aegilops accessions under the two toxic B treatments can be observed in Figures 3I,J. In general, B toxicity tolerance in plants is considered to be associated with its capacity to maintain low B concentrations in the tissues (Cartwright et al., 1986; Nable, 1988; Nable et al., 1990, 1997; Moody et al., 1993; Hayes and Reid, 2004; Rehman et al., 2006; Sutton et al., 2007; Ghaffari Nejad et al., 2015). However, this argument has been rejected by a number of studies where the differences in the behavior toward B toxicity are not associated with B concentration in the tissues (Mahalakshmi et al., 1995; Yau et al., 1995; Torun et al., 2002; Brdar-Jokanovic, 2020). Moreover, tolerant genotypes with higher SB concentrations have also been determined (Torun et al., 2006), and this tolerance is attributed to the plant’s own ability to tolerate high B concentrations (Babaoğlu et al., 2004).

The decreased accumulation of B in shoot tissues is partially attributed to the efflux of B from the roots, which is regulated by the increased expression of a gene encoding the B efflux transporter in roots (Hayes and Reid, 2004; Sutton et al., 2007). Reid and Fitzpatrick (2009) demonstrated that the upregulation of the same gene in leaves, and consequently the B efflux led to the redistribution of B from the intracellular phase to the apoplast, which is comparatively less susceptible to B toxicity. This can be a reason for a poor correlation between SB concentrations and B toxicity symptoms in leaves. However, still, a deep understanding of the mechanism behind this tolerance is required and should be studied in detail focusing on these tolerant genotypes.

### Genetic Dissimilarity Among the Aegilops Accessions Based on the Response Toward B Toxicity Stress

Large variations in terms of root and shoot growth parameters have been observed in the studied Aegilops accessions under highly toxic B growth conditions (Figures 3A–D and Supplementary Figures 1A,B). A similar level of genetic variation in root-shoot growth parameters was observed in previous studies on durum and bread wheat genotypes (Yau et al., 1995; Jamjod, 1996; Kalayci et al., 1998; Torun et al., 2006). Such differences in the response of Aegilops accessions toward B toxicity should be utilized to develop B-tolerant prebreeding material. The genetic profile of the Aegilops accessions can be a major contributor to these variations in response to B toxicity stress. Interestingly, both the genotypes identified as tolerant were tetraploid with the genome UUMM. Moreover, as all the tetraploid genotypes in Cluster 1 consist of the U genome (either along with C or M genome), it might be mis-concluded that the U genome has some contributions in providing B toxicity tolerance. However, it cannot be considered as a favorable conclusion because other tetraploid genotypes containing the U genome are also present in Clusters 2 and 3, which did not show B toxicity tolerance symptoms. Moreover, *A. umbellulata* that only contains the U genome did not show any symptoms of B toxicity tolerance. Thus, based on the results, it can be concluded that B toxicity tolerance in the experiment was not correlated to any ploidy or species but was adhered to specific genotypes.

## Conclusion

In summary, the effect of B toxicity stress on the growth of 19 Aegilops accessions belonging to 6 different species was explored to identify the level of genetic variability in their tolerance to high B toxicity. Our findings suggested that (i) B toxicity stress had a more conclusive impact on growth parameters as compared to MDA and proline content, (ii) The impact of B toxicity stress was more on shoots as compared to roots, (iii) Some of the Aegilops accessions may have greater levels of B toxicity tolerance as compared to the existing B-tolerant wheat cultivars, and the mechanisms providing them the tolerance needs to be thoroughly studied. The two accessions, Ab2 (*A. biuncialis* accession TGB 026219) and Ac4 (*A. columnaris* accession TGB 000107), were considered to be tolerant in this study can be employed for developing the introgression lines or a pre-breeding material for B tolerance-based breeding programs, (iv) B tolerance level is not always associated with the lower SB accumulation. The accessions with the higher RB and SB accumulation can also be tolerant to toxic B levels. This strengthens the argument of the existence of some additional mechanisms other than the B efflux from roots. The redistribution mechanism of B in leaves suggested by Reid and Fitzpatrick (2009) can be taken into account as well. However, it needs to be confirmed by estimating the B concentration of intracellular tissues and apoplast of leaves separately. More detailed research based on antioxidant and molecular analyses is required to understand the other underlying mechanisms of B toxicity tolerance, and the two Aegilops accessions identified as tolerant in this study can be preferred for such further research.

## Data Availability Statement

The original contributions presented in the study are included in the article/Supplementary Material, further inquiries can be directed to the corresponding author.

## Author Contributions

MKK and AP conceived, wrote, and edited the manuscript. MKK, AP, MH, ZZA, MO, AHO, FE, and MRO conducted the experiment and analysis. MKK arranged Figure 1, which comprises the original spike pictures of all the studied Aegilops accessions. FG, AT, and SG made an intellectual contribution to the manuscript. All authors have read and agreed to the content.

## Conflict of Interest

The authors declare that the research was conducted in the absence of any commercial or financial relationships that could be construed as a potential conflict of interest.

## Publisher’s Note

All claims expressed in this article are solely those of the authors and do not necessarily represent those of their affiliated organizations, or those of the publisher, the editors and the reviewers. Any product that may be evaluated in this article, or claim that may be made by its manufacturer, is not guaranteed or endorsed by the publisher.
