# Supplementary material for: Variability in Physiological Traits Reveals Boron Toxicity Tolerance in Aegilops Species
Source: Front Plant Sci. 2021 Oct 29;12:736614. doi: 10.3389/fpls.2021.736614 (PMC8585849; doi:10.3389/fpls.2021.736614)
Supplement: Supplementary file 1 [file Data_Sheet_1.docx]

Supplementary Material

Supplementary Table S1: Means of the RL (root length), SL (Shoot length), RFW (root fresh weight), SFW (shoot fresh weight), RDW (root dry weight), and SDW (shoot dry weight) of 19 Aegilops accessions and the B tolerant check cultivar, Bolal 2973 grown under Control (3.1 μM B), toxic B (1 mM B) and highly toxic B (10 mM B).

| **Code** | **Shoot Length** | | | **Root Length** | | | **Shoot Fresh Weight** | | | **Root Fresh Weight** | | | **Shoot Dry Weight** | | | **Root Dry Weight** | | |
| --- | --- | --- | --- | --- | --- | --- | --- | --- | --- | --- | --- | --- | --- | --- | --- | --- | --- | --- |
|  | **Control** | **1 mM** | **10 mM** | **Control** | **1 mM** | **10 mM** | **Control** | **1 mM** | **10 mM** | **Control** | **1 mM** | **10 mM** | **Control** | **1 mM** | **10 mM** | **Control** | **1 mM** | **10 mM** |
| **Ab1** | 21.67 | 21.50 | 21.33 | 9.50 | 10.50 | 6.67 | 0.21 | 0.22 | 0.17 | 0.08 | 0.06 | 0.04 | 0.030 | 0.033 | 0.032 | 0.009 | 0.008 | 0.007 |
| **Ab2** | 19.67 | 20.17 | 22.33 | 9.50 | 11.83 | 8.33 | 0.21 | 0.26 | 0.20 | 0.08 | 0.11 | 0.06 | 0.027 | 0.035 | 0.039 | 0.009 | 0.011 | 0.008 |
| **Ab3** | 23.33 | 23.00 | 23.00 | 10.33 | 9.83 | 5.50 | 0.26 | 0.22 | 0.19 | 0.10 | 0.07 | 0.05 | 0.034 | 0.030 | 0.033 | 0.010 | 0.009 | 0.007 |
| **Ac1** | 26.50 | 25.00 | 22.83 | 35.50 | 35.50 | 16.33 | 0.39 | 0.41 | 0.27 | 0.25 | 0.24 | 0.09 | 0.043 | 0.046 | 0.040 | 0.015 | 0.014 | 0.008 |
| **Ac2** | 24.17 | 23.17 | 20.00 | 11.33 | 10.83 | 6.83 | 0.34 | 0.38 | 0.17 | 0.11 | 0.14 | 0.04 | 0.040 | 0.043 | 0.033 | 0.012 | 0.012 | 0.007 |
| **Ac3** | 23.50 | 20.67 | 19.67 | 23.17 | 30.00 | 10.67 | 0.23 | 0.21 | 0.12 | 0.10 | 0.11 | 0.02 | 0.026 | 0.024 | 0.018 | 0.006 | 0.006 | 0.002 |
| **Ac4** | 20.83 | 22.67 | 23.67 | 9.67 | 9.33 | 7.33 | 0.23 | 0.29 | 0.22 | 0.08 | 0.11 | 0.07 | 0.029 | 0.036 | 0.036 | 0.009 | 0.009 | 0.008 |
| **Ac5** | 24.17 | 26.33 | 22.17 | 29.67 | 28.33 | 16.33 | 0.27 | 0.28 | 0.14 | 0.16 | 0.16 | 0.08 | 0.031 | 0.033 | 0.024 | 0.011 | 0.010 | 0.006 |
| **As1** | 24.67 | 24.00 | 23.17 | 25.00 | 20.33 | 14.83 | 0.15 | 0.14 | 0.06 | 0.09 | 0.18 | 0.02 | 0.018 | 0.018 | 0.010 | 0.007 | 0.005 | 0.002 |
| **As2** | 21.67 | 24.33 | 21.25 | 17.33 | 23.67 | 14.50 | 0.14 | 0.15 | 0.08 | 0.07 | 0.07 | 0.03 | 0.018 | 0.018 | 0.014 | 0.006 | 0.005 | 0.003 |
| **Al1** | 20.50 | 22.50 | 22.00 | 15.83 | 20.67 | 14.33 | 0.10 | 0.12 | 0.07 | 0.05 | 0.05 | 0.02 | 0.012 | 0.013 | 0.010 | 0.003 | 0.003 | 0.002 |
| **Al2** | 27.00 | 31.67 | 21.83 | 25.33 | 28.33 | 15.17 | 0.27 | 0.25 | 0.09 | 0.17 | 0.11 | 0.03 | 0.029 | 0.028 | 0.015 | 0.009 | 0.007 | 0.003 |
| **At1** | 22.33 | 17.83 | 18.00 | 22.67 | 22.50 | 13.67 | 0.26 | 0.19 | 0.14 | 0.15 | 0.09 | 0.05 | 0.030 | 0.021 | 0.020 | 0.008 | 0.005 | 0.004 |
| **At2** | 21.50 | 21.83 | 18.00 | 22.50 | 29.17 | 16.00 | 0.22 | 0.22 | 0.17 | 0.11 | 0.09 | 0.06 | 0.022 | 0.023 | 0.021 | 0.006 | 0.006 | 0.005 |
| **At3** | 24.17 | 21.00 | 20.00 | 25.33 | 21.67 | 10.83 | 0.27 | 0.18 | 0.13 | 0.12 | 0.06 | 0.02 | 0.034 | 0.019 | 0.020 | 0.008 | 0.004 | 0.003 |
| **At4** | 23.83 | 21.50 | 23.67 | 35.67 | 18.83 | 19.67 | 0.34 | 0.30 | 0.26 | 0.21 | 0.17 | 0.07 | 0.041 | 0.042 | 0.032 | 0.012 | 0.011 | 0.004 |
| **Au1** | 19.00 | 19.33 | 17.33 | 26.00 | 21.50 | 12.83 | 0.19 | 0.17 | 0.13 | 0.14 | 0.10 | 0.05 | 0.026 | 0.022 | 0.024 | 0.008 | 0.007 | 0.005 |
| **Au2** | 21.67 | 23.00 | 21.83 | 27.17 | 29.17 | 15.67 | 0.25 | 0.29 | 0.16 | 0.27 | 0.24 | 0.07 | 0.033 | 0.038 | 0.028 | 0.015 | 0.015 | 0.006 |
| **Au3** | 18.67 | 18.50 | 16.50 | 28.50 | 34.50 | 15.00 | 0.24 | 0.26 | 0.14 | 0.21 | 0.27 | 0.06 | 0.029 | 0.034 | 0.025 | 0.012 | 0.015 | 0.006 |
| **Bolal** | 39.17 | 31.00 | 28.33 | 26.17 | 22.00 | 17.50 | 0.50 | 0.41 | 0.38 | 0.14 | 0.09 | 0.07 | 0.053 | 0.042 | 0.053 | 0.011 | 0.009 | 0.007 |

Supplementary Table S2: Means of the RMDA (root malondialdehyde), SMDA (shoot malondialdehyde), RPro (root proline), SPro (shoot proline), RB (root boron accumulation), and SB (shoot boron accumulation) of 19 Aegilops accessions and the B tolerant check cultivar, Bolal 2973 grown under Control (3.1 μM B), toxic B (1 mM B) and highly toxic B (10 mM B).

| **Code** | **Shoot MDA** | | | **Root MDA** | | | **Shoot Proline** | | | **Root Proline** | | | **Shoot B accumulation** | | | **Root B accumulation** | | |
| --- | --- | --- | --- | --- | --- | --- | --- | --- | --- | --- | --- | --- | --- | --- | --- | --- | --- | --- |
|  | **Control** | **1 mM** | **10 mM** | **Control** | **1 mM** | **10 mM** | **Control** | **1 mM** | **10 mM** | **Control** | **1 mM** | **10 mM** | **Control** | **1 mM** | **10 mM** | **Control** | **1 mM** | **10 mM** |
| **Ab1** | 17.15 | 31.46 | 32.53 | 7.30 | 9.48 | 10.93 | 0.032 | 0.032 | 0.045 | 0.032 | 0.035 | 0.048 | 0.61 | 16.59 | 83.01 | 0.33 | 1.52 | 16.97 |
| **Ab2** | 20.60 | 26.04 | 30.87 | 8.13 | 9.21 | 11.81 | 0.035 | 0.042 | 0.066 | 0.022 | 0.038 | 0.045 | 0.51 | 18.51 | 131.24 | 0.07 | 0.21 | 3.78 |
| **Ab3** | 12.25 | 25.01 | 31.57 | 12.21 | 18.07 | 15.46 | 0.061 | 0.082 | 0.089 | 0.029 | 0.034 | 0.049 | 0.53 | 15.93 | 105.71 | 0.11 | 1.87 | 17.80 |
| **Ac1** | 7.60 | 9.83 | 15.60 | 5.67 | 7.47 | 11.43 | 0.019 | 0.034 | 0.018 | 0.028 | 0.050 | 0.064 | 0.81 | 35.02 | 101.48 | 0.32 | 2.03 | 3.26 |
| **Ac2** | 21.55 | 24.89 | 49.18 | 9.11 | 10.98 | 16.78 | 0.063 | 0.070 | 0.084 | 0.018 | 0.038 | 0.040 | 0.55 | 13.15 | 130.97 | 0.06 | 0.64 | 2.36 |
| **Ac3** | 17.07 | 28.72 | 31.98 | 7.94 | 8.57 | 11.52 | 0.011 | 0.060 | 0.050 | 0.014 | 0.028 | 0.024 | 0.55 | 10.64 | 66.36 | 0.10 | 0.39 | 1.62 |
| **Ac4** | 14.31 | 20.41 | 27.15 | 12.62 | 13.27 | 14.60 | 0.032 | 0.062 | 0.083 | 0.040 | 0.062 | 0.071 | 0.56 | 16.27 | 295.62 | 0.14 | 2.24 | 15.46 |
| **Ac5** | 11.40 | 15.50 | 22.46 | 8.02 | 12.10 | 14.17 | 0.024 | 0.043 | 0.055 | 0.037 | 0.050 | 0.048 | 0.71 | 18.54 | 52.25 | 0.14 | 0.60 | 1.63 |
| **As1** | 14.31 | 14.25 | 26.35 | 6.99 | 9.15 | 9.39 | 0.025 | 0.026 | 0.037 | 0.025 | 0.021 | 0.044 | 0.28 | 4.88 | 49.72 | 0.08 | 0.16 | 1.38 |
| **As2** | 7.28 | 17.50 | 18.21 | 7.04 | 7.20 | 10.32 | 0.016 | 0.040 | 0.051 | 0.018 | 0.038 | 0.043 | 0.30 | 4.97 | 30.52 | 0.15 | 0.55 | 1.47 |
| **Al1** | 21.32 | 22.12 | 24.77 | 6.40 | 8.57 | 10.12 | 0.024 | 0.021 | 0.029 | 0.017 | 0.033 | 0.033 | 0.45 | 4.56 | 42.65 | 0.03 | 0.27 | 0.75 |
| **Al2** | 14.00 | 15.85 | 35.81 | 6.78 | 8.26 | 8.05 | 0.016 | 0.054 | 0.060 | 0.025 | 0.032 | 0.027 | 0.60 | 13.73 | 55.60 | 0.13 | 0.67 | 0.98 |
| **At1** | 8.27 | 18.95 | 22.23 | 5.67 | 8.50 | 15.50 | 0.017 | 0.034 | 0.037 | 0.043 | 0.049 | 0.083 | 0.40 | 7.84 | 43.57 | 0.05 | 0.25 | 1.95 |
| **At2** | 18.57 | 22.99 | 35.22 | 6.71 | 7.78 | 8.86 | 0.018 | 0.019 | 0.028 | 0.013 | 0.053 | 0.024 | 0.51 | 7.78 | 52.52 | 0.08 | 0.50 | 1.46 |
| **At3** | 17.60 | 25.80 | 37.43 | 5.60 | 8.20 | 13.27 | 0.017 | 0.018 | 0.051 | 0.021 | 0.047 | 0.069 | 0.77 | 8.52 | 72.95 | 0.22 | 0.48 | 5.91 |
| **At4** | 9.60 | 13.33 | 19.30 | 7.13 | 12.70 | 16.30 | 0.009 | 0.018 | 0.011 | 0.060 | 0.060 | 0.085 | 0.63 | 23.45 | 73.71 | 0.13 | 1.30 | 3.31 |
| **Au1** | 16.53 | 27.26 | 28.23 | 6.17 | 7.13 | 11.43 | 0.015 | 0.039 | 0.028 | 0.026 | 0.038 | 0.058 | 0.33 | 9.89 | 74.23 | 0.13 | 0.48 | 3.34 |
| **Au2** | 14.30 | 24.40 | 28.23 | 6.07 | 9.63 | 15.40 | 0.024 | 0.028 | 0.028 | 0.037 | 0.043 | 0.064 | 0.30 | 19.35 | 106.75 | 0.08 | 0.92 | 3.97 |
| **Au3** | 10.20 | 32.20 | 17.67 | 5.67 | 6.39 | 11.27 | 0.029 | 0.049 | 0.015 | 0.059 | 0.078 | 0.047 | 0.46 | 19.95 | 84.30 | 0.19 | 1.49 | 2.59 |
| **Bolal** | 10.05 | 11.33 | 15.04 | 7.63 | 8.86 | 8.38 | 0.024 | 0.026 | 0.074 | 0.008 | 0.013 | 0.024 | 1.18 | 17.31 | 129.68 | 0.52 | 1.04 | 6.04 |

**Supplementary Figures**

S1a)
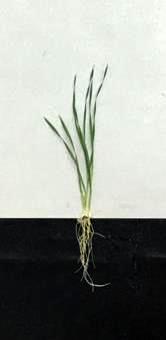
 S1b)
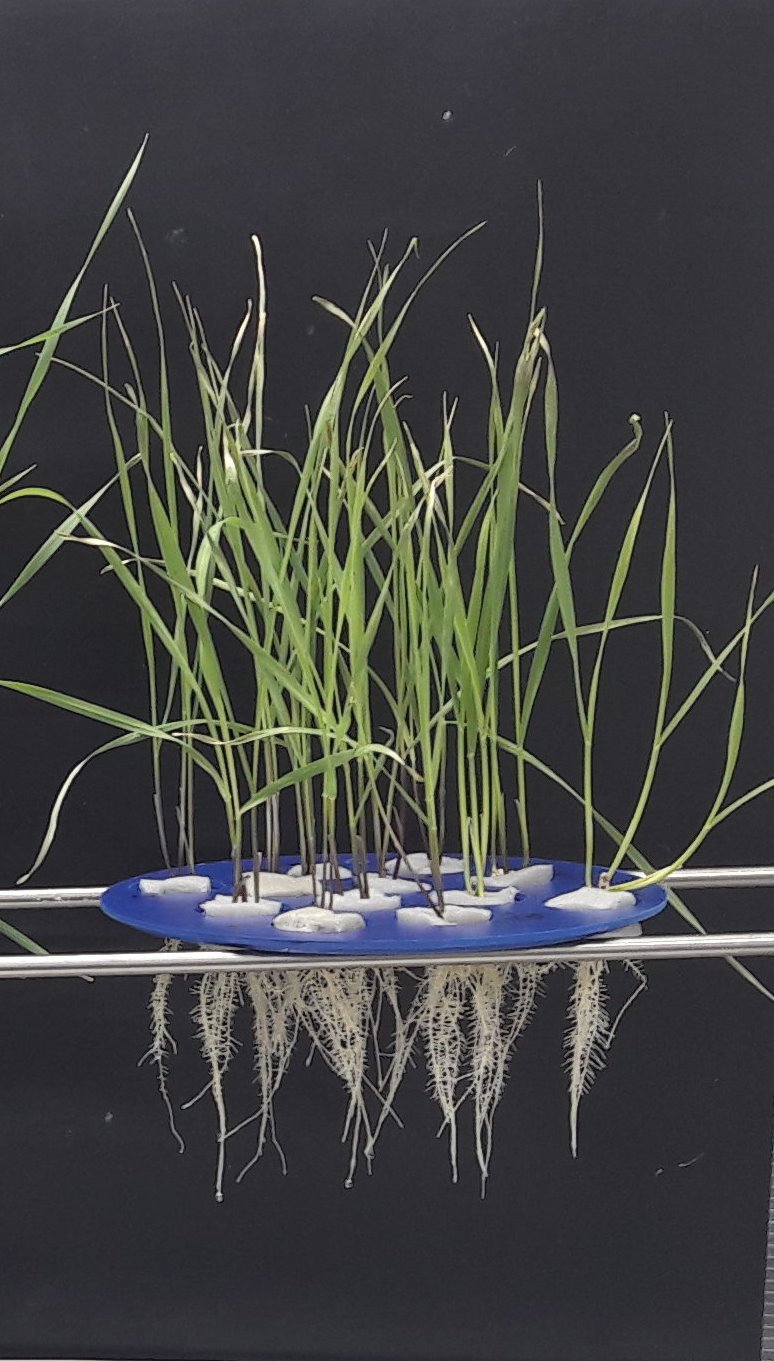


Supplementary Figure S1. Pictures showing the growth stage of the (a) single plant and (b) different plants grown in hydroponic system just before the harvest (7^th^ day after B treatment).

S2a)


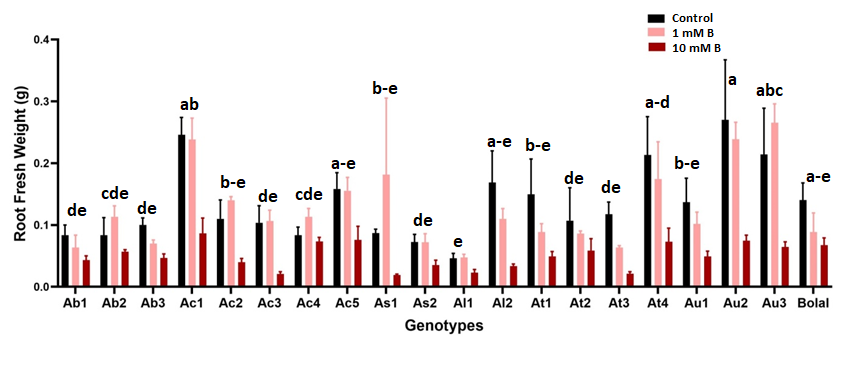


S2b)


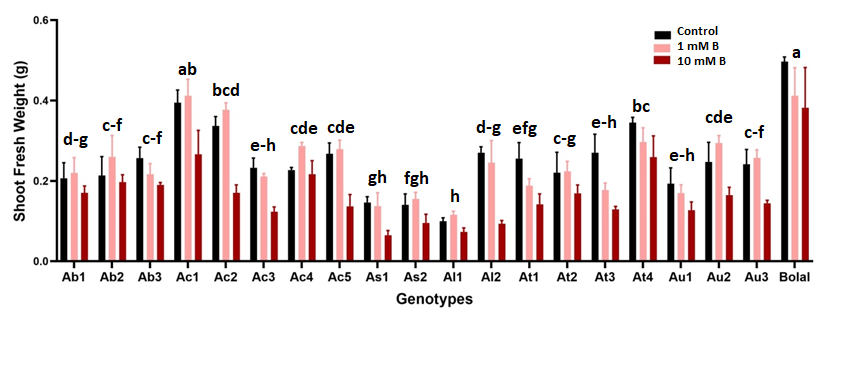


Supplementary Figure S2. Variability in the (a) root fresh weight and (b) shoot fresh weight of 19 Aegilops accessions and the B tolerant check cultivar, Bolal 2973 grown under Control (3.1 μM B), toxic B (1 mM B) and highly toxic B (10 mM B). Data represent means ± SE. Tukey’s pairwise comparison using the general linear model was employed to distinguish any significant differences among the experimental genotypes. Genotypes that do not share a letter are significantly different.


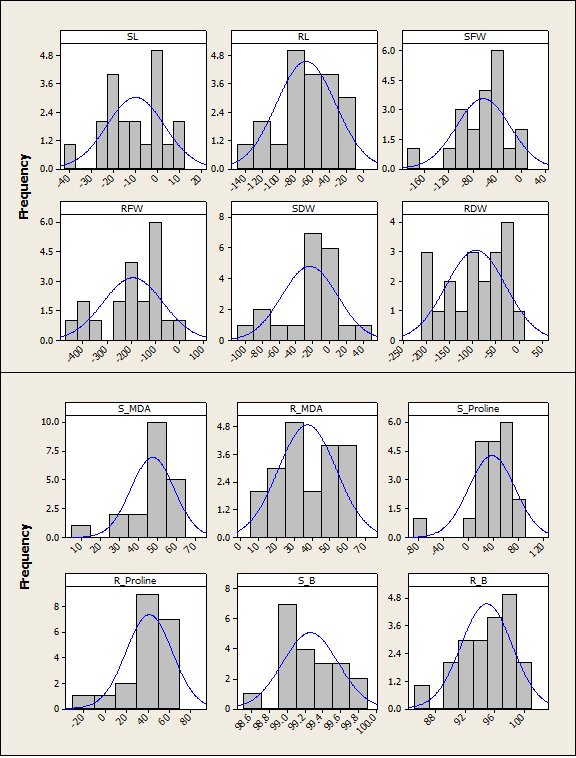


Supplementary Figure S3. The frequency distribution of 19 Aegilops accessions and the B tolerant check cultivar, Bolal 2973 based on the percentage changes under 10 mM B treatment as compared to the control for all measured traits including shoot length (SL), root length (RL), shoot fresh weight (SFW), root fresh weight (RFW), shoot dry weight (SDW), root dry weight (RDW), shoot malondialdehyde (S_MDA), root malondialdehyde (R_MDA), shoot proline (S_Proline), root proline (R_Proline), shoot B accumulation (S_B) and root B accumulation (R_B). The normal graph was plotted for all the measured traits.
